# Changes in Transformational Leadership and Empirical Quality Outcomes in a Finnish Hospital over a Two-Year Period: A Longitudinal Study

**DOI:** 10.1155/2014/218069

**Published:** 2014-06-10

**Authors:** Raija Mäntynen, Katri Vehviläinen-Julkunen, Pirjo Partanen, Hannele Turunen, Merja Miettinen, Tarja Kvist

**Affiliations:** ^1^Department of Nursing Science, University of Eastern Finland, P.O. Box 1627, 70211 Kuopio, Finland; ^2^Kuopio University Hospital, P.O. Box 1777, 70211 Kuopio, Finland; ^3^University of Tampere, 33014 Tampere, Finland

## Abstract

This paper describes the changes in transformational leadership and quality outcomes that occurred between 2008 and 2011 in a Finnish university hospital that is aiming to meet the Magnet standards. Measurements were conducted in 2008-2009 and subsequently in 2010-2011 by surveying nursing staff and patients. Nursing staff were surveyed using web-based surveys to collect data on transformational leadership (*n*_1_ = 499, *n*_2_ = 498) and patient safety culture (*n*_1_ = 234, *n*_2_ = 512) and using both postal and web-based surveys to gather information on job satisfaction (*n*_1_ = 1176, *n*_2_ = 779). Questionnaires were used to collect data on care satisfaction from patients (*n*_1_ = 678, *n*_2_ = 867). Transformational leadership was measured using the 54-item TLS, job satisfaction with the 37-item KUHJSS, patient safety culture with the 42-item HSPSC, and patient satisfaction using the 42-item RHCS questionnaire. Transformational leadership, which was the weakest area, was at the same level between the two measurement occasions. Job satisfaction scores increased between 2008 and 2010, although they were generally excellent in 2008. The scores for nonpunitive responses to errors and events reported were also higher in the 2010-2011 surveys. The highest empirical outcome scores related to patient satisfaction. The project and the development initiatives undertaken since 2008 seem to have had positive effects on empirical quality outcomes.

## 1. Introduction

The Attractive and Safe (At Safe) Hospital project [1] was initiated in 2006 with the aim of bringing a Finnish university hospital into compliance with the Magnet standards [2]. The project received national funding during the period between 2009 and 2011, which significantly increased its scope and made it possible to implement some development initiatives.

The Magnet designation of the American Nurses Credentialing Center (ANCC) is the highest international standard for nursing excellence. The new Magnet Model was presented in 2008 and is based on five components: transformational leadership; structural empowerment; exemplary professional practice; new knowledge, innovations, and improvements; and empirical quality outcomes [2–5]. To evaluate the progress of the At Safe project, we studied its effects on two of these components: transformational leadership and empirical quality outcomes. Specific quality outcomes of interest included job satisfaction among nurses, factors relating to patient safety culture, and patient satisfaction. These components were selected based on previous investigations conducted within our research group. In addition, nurse leaders had identified aspects of transformational leadership and empirical quality outcomes as being in need of improvement. Baseline information on these components was gathered in 2008-2009 via a series of surveys and questionnaires [5]. Follow-up investigations using the same surveys and questionnaires were then conducted in 2010-2011. Here, we describe the changes in the survey responses between the years 2008 and 2011 and some related developments that are currently under way.

Sanders et al. [6] have described initiatives aimed at establishing healthy and satisfying working environments for nurses. Interventions that have been evaluated for this purpose include the introduction of uninterrupted meal breaks, physician-nurse collaborations, provision of resources to ensure adequate staffing, and access to evidence-based practice (EBP) courses. It has also been demonstrated that change is most likely to occur when strongly supported by the nursing unit's leadership team. However, this raises a question: what is the best way of coaching nurse leaders to encourage such change? According to the studies of Poss et al. [7] and Kelly et al. [8], the nurse leaders with more leadership training are able to motivate their staff for the change. In addition, the higher educational degree of nurse leaders can increase their ability to empower the nurses in changing health care environment [8].

Leadership development programs [9] and formal training [8] have been used to prepare nurse leaders to meet health care challenges. A joint academic-service nursing leadership journal club was shown to be effective at increasing awareness of nursing leadership research and promoting leadership [10]. The existence of strong collaborations between nurse leaders and nursing staff may also promote shared governance, exemplified by things such as nurse participation in practice council activities. Such collaborations empower nursing staff and increase their job satisfaction [6, 11, 12].

Nurse managers are vital in promoting job satisfaction among nurses [13, 14]. Transformational leadership style of nurse managers influences positively the job satisfaction of the staff nurses [15]. In one notable study, nursing staff who experienced fewer instances of missed nursing care reported higher levels of job satisfaction [16]. Evidence-based practice councils can improve the quality of care, job satisfaction among nurses, and leadership in hospitals [11].

Nurse managers also play important roles in the development of a strong patient safety culture [17]. Units with high and low LMX (leader-member exchange) scores were found to differ significantly with respect to diverse factors, including supervisor safety expectations, organizational learning-continuous improvement, total communication, feedback and communication about errors, and nonpunitive responses to errors [18]. However, nurse managers must support nurses and develop working environments in which nurses can implement high-quality and safe patient care [19]. Benn et al. [20] found that lasting positive changes in patient safety culture can only be achieved if hospitals invest heavily into development programs. In all cases, the first step in the development of a patient safety culture is the reporting of errors when they occur [21].

The patients at the studied hospitals are generally very satisfied with their care [5, 22–26]. Finnish studies conducted since 1990 have shown that patients sometimes feel inadequately informed about various aspects of their treatment and that they are not given opportunities to contribute to their own care [22–24]. Conry et al. [25] showed that there have been relatively few theory-based interventions aimed at improving the quality of care in hospitals. However, interventions aimed at improving the working environment within hospitals may be relatively inexpensive methods of improving quality of care and patient safety [27]. Kooker and Kamikawa [28] have shown that professional development improves patient outcomes. In addition, nurse management was shown to be a significant positive predictor of good or excellent perceived quality of care within a hospital unit [29, 30]. On the other hand, Rozenblum et al. [31] highlighted the importance of structured plans for promoting improvements in patient satisfaction and the engagement of nurses and physicians within hospitals.

The aim of this study was to describe the changes in transformational leadership and empirical quality outcomes (specifically, job satisfaction among nurses, patient safety culture, and patient satisfaction) that occurred in a Finnish university hospital between the years 2008 and 2011 during the course of a project that was intended to bring the hospital into compliance with the Magnet standards.

## 2. Methods

A longitudinal study with both descriptive and quantitative aspects was conducted. The studied university hospital has 770 beds and around 2700 nursing staff. It provides highly specialized health care within the area it serves.

### 2.1. Interventions Aimed at Improving Transformational Leadership, Job Satisfaction, Patient Safety Culture, and Patient Satisfaction That Have Been Implemented Since 2008

Development work aimed at improving transformational leadership, job satisfaction, patient safety culture, and patient satisfaction within the hospital was initiated in 2008. Some representative interventions are described below.

*Transformational Leadership.* A Nursing Research Council was established in the hospital in 2008. Its purpose is to support nurse leaders by increasing the visibility of nursing research and evidence-based practice. Second, a long-lasting evidence-based leadership course for nurse leaders working within the hospital's district (*n* = 47) was offered between September 2010 and May 2011. The course employed a range of learning methods including lectures, group work, and assignments in web-based learning environments. In addition, the participating nurse leaders and their units were informed of the results gathered during the initial transformational leadership survey.

Job satisfaction was promoted via a systematic orientation to nursing work based on web-based training. In addition, all participants were informed of the results gathered during the initial job satisfaction survey, with each unit receiving individualized results based on the responses of its participating members. The Nursing Research and Evidence-Based Practice councils also contributed significantly to the promotion of job satisfaction.

The intervention aimed at improving patient safety culture was based on the HaiPro error-reporting system [32] and the provision of systematic medication training for staff. HaiPro is a national electronic reporting system that the studied university hospital was among the first in Finland to adopt.

One fixed intervention which aimed to advance patient satisfaction was an evidence-based practice training project involving nursing staff (*n* = 38) around the hospital from different units. It was conducted during 2009 and 2010. The participating nurses produced written assignments aimed at developing nursing care in their units. Three main areas of focus were selected and they concentrated on heart failure patients, breast cancer, and depression patients. These patient groups form a big number of the cared patients. In addition, a model for preventing patients from falling was introduced into practice within the university hospital.

### 2.2. Sampling

The data collection procedure used in this work was described at length in our previous article [5]. Data were collected on two occasions: baseline data were collected in 2008-2009 and follow-up data were gathered in 2010-2011 (Table 1).

In 2008, a survey on transformational leadership was sent out to 1965 nursing staff, with a response rate of 25% (*n* = 499). In 2010, 2105 questionnaires were sent out and 498 were returned, giving a response rate of 24%. In 2008, a survey on job satisfaction was sent by mail to 2070 nursing staff and nursing leaders, with a response rate of 57% (*n* = 1176); in 2010, 779 of 2641 nursing staff members (29%) completed a web-based version of the same survey. In 2008, a survey on patient safety culture was sent out to 1802 nursing staff with 234 responses being received (corresponding to a rather low response rate of 13%). A slightly higher response rate of 23% was achieved for the same survey in 2011 (512 of 2225 contacted nursing staff and nursing leaders responded). Each member of staff who was asked to complete a survey was sent one reminder by e-mail for every survey they had been given (Table 1).

Patient data were collected from responses to questionnaires posted in November of 2008 to the home addresses of patients who had received care in September of 2008. The randomized sample included approximately 10% of all patients cared for in every studied inpatient and outpatient unit during the specified months. The survey was sent to 1773 patients with a response rate of 38% (*n* = 678). In November 2010, questionnaires were given directly to patients as they were leaving the unit. 2315 questionnaires were handed out to patients and 867 were returned by post to the researchers, giving a response rate of 37%. No reminders were sent out in either year (Table 1).

### 2.3. Survey Instruments and Reliability

The survey instruments are presented in Table 2. A Transformational Leadership Scale was developed in the course of this work, based on literature reviews with input from expert panels and pilot studies [5, 33]. The Transformational Leadership Scale consists of 54 items divided into five subscales. The possible responses to each item ranged from 1 (“strongly disagree”) to 5 (“strongly agree”). Mean scores were computed for the responses to the items in each subscale and an overall scale score was computed from the mean scores of the subscales. The reliability of the instrument was good, yielding Cronbach's *α* values of 0.909 to 0.968 (for 2008) and 0.916 to 0.950 (for 2010).

The Kuopio University Hospital Job Satisfaction Scale (KUHJSS) [34] was also developed during this research project. The KUHJSS was founded on literature reviews and developed with input from expert panels and pilot studies. It comprises 37 items divided into seven subscales that were delineated on the basis of exploratory factor analysis. The possible responses to each item ranged from 1 (“strongly disagree”) to 5 (“strongly agree”). Mean scores were computed for the responses to the items in each subscale and an overall scale score was computed from the mean scores of the subscales. The reliability of the instrument was evaluated by calculating its Cronbach *α* values, which ranged from 0.641 to 0.916 in 2008 and 0.652 to 0.918 in 2010 (Table 2).

The patient safety culture at the studied hospital was characterized using the Hospital Survey on Patient Safety Culture (HSPSC), which was developed and tested by the Agency for Healthcare Research and Quality (AHRQ) of the Department of Health and Human Services in the United States. The HSPSC instrument is freely available for use by all researchers. Before being used in this work, it was translated from English into Finnish and then independently back to English, to verify the accuracy of the Finnish translation. The scale includes 42 items that measure 12 subscales of patient safety culture. The possible responses to each item range from 1 (“strongly disagree”) to 5 (“strongly agree”). Mean scores were computed for the responses to the items in each subscale and an overall scale score was computed from the mean scores of the subscales. Cronbach's *α* values for the HSPSC instrument in this study ranged from 0.481 to 0.804 in 2008 and 0.449 to 0.802 in 2011 (Table 2). For comparative purposes, the values reported by [18] Thompson et al. ranged from 0.720 to 0.830.

Patient satisfaction was characterized using the Revised Humane Caring Scale (RHCS) [5]. The RHCS is a revised version of the Humane Caring Scale [22], which was developed to measure the quality of care provided by the staff of a hospital as a whole [23]. Its performance was evaluated in a pilot test before its use in the main study. Both of these instruments were developed in part by the authors of this paper (Table 2).

The original Humane Caring Scale (HCS) was developed at Kuopio University Hospital in the early 1990s and has been widely used to characterize patients' opinions on the quality of their care. In this study, the RHCS was used to measure patient satisfaction. The HCS has been used in several previous studies and has recently been revised again [23, 24]. The 42 items of the RHCS are divided into six subscales [5]. The possible responses to each item range from 1 (“strongly disagree”) to 5 (“strongly agree”). Mean scores were computed for the responses to the items in each subscale and an overall scale score was computed from the mean scores of the subscales. The reliability of the instrument was good, yielding Cronbach's *α* values of 0.775 to 0.946 in 2008 and 0.776 to 0.950 in 2010 (Table 2).

### 2.4. Analysis

The survey responses were analyzed using descriptive statistics, exploratory factor analysis, reliability analysis (Cronbach's *α*), and nonparametric (Mann-Whitney) tests to assess differences between years. All statistical analyses were performed using SPSS version 19.0 for Windows (SPSS Inc., Chicago, IL, USA).

We set the target level for transformational leadership and the empirical quality outcomes at excellent [5], which is defined by mean scores of 4 or more on the corresponding instruments. This is comparable to the criteria applied in the Magnet assessment scales, where a score of 4 is defined as meeting the Magnet standards [2].

### 2.5. Ethical Considerations

The study design was reviewed and approved by the Research Ethical Committee of the Northern Savo Hospital District (Permission numbers 46/2007 and 66§/2010). In addition, research permission was given by the chief executive medical directors, chief nursing officers, and personnel managers of the university hospital. In each case, the survey documents and questionnaires included the researchers' contact details and information about the study. Participation was voluntary and anonymous.

## 3. Results

### 3.1. Nursing Staff Characteristics

Most of the nursing staff at the hospital were women. In 2008, 35% of respondents to the leadership survey and 33% of respondents to the job satisfaction survey were 41 to 50 years old. Individuals with more than 21 years of work experience in their current unit accounted for 16% of all respondents to the leadership survey, 18% in the job satisfaction survey, and 13% in the patient safety culture survey. Most of the nursing staff (78%) were employed on a permanent basis.

In 2010, 34% of respondents to the leadership survey and 31% of respondents to the job satisfaction were 41 to 50 years old. Individuals with more than 21 years of work experience in their current unit accounted for 12% of all respondents to the leadership survey, 20% in the job satisfaction survey, and 19% in the patient safety culture survey (in 2011). Most of the nursing staff (79% for the leadership survey and 82% for the job satisfaction survey**)** were employed on a permanent basis.

### 3.2. Patient Characteristics

Over half (60%) of the patients were women in both years. In 2008-2009, the average patient age was 55 years (ranging from 16–90). The average length of stay at the hospital was 5.9 days (ranging from 1 to 80 days). Nearly half (49%) of the patients had a vocational education, 24% had no education, and 11% had a university degree.

In 2010, the average age of the patients was 54 years (ranging from 16 to 89). The average length of stay at the hospital was 6.0 days (ranging from 1 to 150). Half (50%) of the patients had a vocational degree, 21% had no education, and 9% had a university degree.

### 3.3. Changes in Transformational Leadership, Job Satisfaction, Patient Safety Culture, and Patient Satisfaction between 2008-2009 and 2010-2011

#### 3.3.1. Transformational Leadership

In 2008, the mean total transformational leadership score was 3.34, while that in 2010 was 3.39. The mean scores for the transformational leadership subscales were generally somewhat higher, with the exception of that for the leadership of nursing directors in 2010. The management of the nursing process was considered to be at the same level (*M* = 3.43) in both surveys, with SD values of 0.87 for 2008 and 0.88 for 2010. All of the mean scores for transformational leadership were below the target level of 4 (Table 3).

#### 3.3.2. Job Satisfaction

The scores for most of the job satisfaction subscales increased between 2008 and 2010, with the 2010 values ranging from 3.16 (SD = 0.76) to 4.27 (SD = 0.60). The sole exception was the working environment subarea, for which the mean score was 3.28 in both cases (SD = 0.88 for 2008 and 0.95 for 2010). The changes were statistically significant (*P* < 0.05) for all subscales other than working welfare and the working environment. The mean scores for the “motivating factors of work” and “working welfare” subscales were both above 4 in 2010 (Table 3).

#### 3.3.3. Patient Safety Culture

The mean scores for the following patient safety culture subscales increased between 2008-2009 and 2010-2011: overall perception of patient culture, error-related feedback and communication, events reported, and nonpunitive responses to errors. The mean scores for these subscales in 2010-2011 ranged from 3.14 (SD = 0.76) to 3.48 (SD = 0.71). However, the mean scores for some subscales declined over the same period: handoffs and transitions (*P* < 0.001), management support for patient safety, staffing, teamwork across units (*P* < 0.001), organizational learning and continuous improvement, communication openness, manager expectations and actions, and teamwork within units. The mean scores for these subscales in 2010-2011 ranged from 3.03 (SD = 0.65) to 3.69 (SD = 0.66). A decline was also observed in the total patient safety score, which was 3.33 (SD = 0.44) in 2010-2011. None of the mean scores for the patient safety culture subscales were above 4 (Table 3).

#### 3.3.4. Patient Satisfaction

High levels of patient satisfaction were recorded in both surveys. The mean scores for two patient satisfaction subscales did not change between 2008-2009 and 2010-2011, namely, cognition of physical needs (*M* = 4.41, SD = 0.83 in 2008-2009 and 0.87 in 2010) and pain and apprehension management (*M* = 4.05, SD = 0.86 in 2008-2009 and 0.89 in 2010). The scores for all of the other subscales increased in the second survey, with mean values ranging from 4.06 (SD = 1.07) to 4.62 (SD = 0.56). The change in the score for the human resources subscale was statistically significant (*P* = 0.023). All of the mean scores in this area were greater than 4 (Table 4).

## 4. Discussion

### 4.1. Discussion of the Results

In this study we investigated the changes that occurred in one hospital over a period of two years using a range of measurements based on the Magnet hospital concept. The results obtained concerning empirical outcomes and transformational leadership showed that the university hospital was a smoothly functioning healthcare organization that exhibits excellence in some aspects [5]. There was mostly positive development in empirical outcomes and transformational leadership. The follow-up period used in this case was relatively short, and there was evidence that development had not occurred in a systematic way. Previous studies have demonstrated a need for a new approach to leadership within the organization [5, 33]. In keeping with this requirement, the survey responses revealed that there had been changes with respect to several aspects of transformational leadership between the two survey periods. However, more intensive interventions, like formal training programs and higher educational degree of nurse leaders [8], would be needed to establish a consistently excellent level of leadership. Making a change from a transactional leadership style to a transformational one requires a change in leadership culture [12, 35]. The introduction of new leadership structures and processes such as nursing councils is one way of establishing this new style of leadership. The participation of nurses in councils is important because it enables them to contribute to decision-making at the organizational level. Evidence-based leadership training is reported to have a positive impact, but it must be available to all leaders. The next goal in bringing the hospital up to the Magnet standard will be to encourage the nursing leadership to make the transition from an experience-based approach to an evidence-based one [36].

The authors considered it important to inform the nursing leaders and staff at the hospital of the study's results because this knowledge may serve as a starting point for change. To this end, it was important to ensure that the findings were readily available and could be discussed at every level within the hospital. The researchers therefore distributed their findings to the leaders of the nursing units, divisions, and the hospital as a whole and encouraged them to share the results with their staff.

The total job satisfaction of the nursing staff was high, with excellent motivation and working welfare. This was considered beneficial for the recruitment of the new staff in areas where it was required. Finnish nurses in general are well educated and highly professional. This was noticed and appreciated by the patients, who reported excellent levels of satisfaction [5, 24]. The relationship between transformational leadership and job satisfaction of nurses would be useful to study [15].

Patient safety culture is a relatively new concept in Finnish health care, although systematic quality projects have been conducted since the 1980s with the aim of highlighting the importance of safety issues and promoting improvement. Nurses should aim to continuously improve the quality of care provided to patients and to ensure that their working processes are understood and promote patient safety. A key challenge for the nursing staff is to adopt new ways of discussing and analyzing questions of patient safety through organizational learning [21, 37].

The hospital exists to serve the needs of patients, so they should have more opportunity to participate in their own care and the general development of the hospital. This can be achieved by inviting them to join the hospital's leadership councils, which is quite a new approach in Finland [38]. Evidence-based practice courses aim to promote patient care, and the adoption of evidence-based practice will ensure that patients receive the best care possible. While the studied hospital is making the transition from experience-based practice to an evidence-based approach, the process will take time.

The results presented herein demonstrate that the interventions that were introduced between the two survey occasions have had meaningful results and should be continued. More interventions would be needed in informal training for the nurse leaders and in participation on the boards and committees. However, it would be useful to complement them with additional theory-based interventions in the future [25].

### 4.2. Limitations of the Study

The interventions that took place between the survey occasions were not implemented systematically or grounded in theory. Their aim was to develop the hospital in a general way and in part through targeted training. In addition, this study was not based on a specific intervention or a quasiexperimental approach, although we did adopt a longitudinal design. Finally, not all of the nursing staff participated in the interventions.

## 5. Conclusions

Improvements were detected in most of the areas considered between the first and second survey periods. Highly motivated and professional staff provide the basis for the excellent nursing care reported by the patients and the nursing staff. However, there is an urgent need for a more transformational approach to nursing leadership within the hospital. To this end, the nurse leaders require training, supervision, and support from their own leaders. Patient safety issues are being increasingly prioritized and targeted for development in the Finnish health care sector. Encouraging the development of a strong patient safety culture throughout individual units and the hospital as a whole will enable further improvements in quality of care.

## Figures and Tables

**Table 1:** Surveys sent outand response rates (*n*, %) in 2008-2009 and 2010-2011.

Survey	2008-2009 Sent	2008-2009 Answers	2008-2009 Response rate	2010-2011 Sent	2010-2011 Answers	2010-2011 Response rate
Transformational leadership (nursing staff)	1965	499	25	2105	498	24
Job satisfaction (nursing staff and nursing leaders)	2070	1176	57	2641	779	29
Patient safety culture (nursing staff and nursing leaders)	1802	234	13	2225	512	23
Patient satisfaction (inpatients and outpatients)	1773	678	38	2315	867	37

**Table 2:** The instruments, subscales, and numbers of variables in the transformational leadership, job satisfaction, patient safety culture, and patient satisfaction surveys.

Instruments	Transformational Leadership Scale (TLS)	Kuopio University Hospital Job Satisfaction Scale (KUHJSS)	Hospital Survey on Patient Safety Culture (HSPSC)	Revised Humane Caring Scale (RHCS)
Subscales (number of variables)	Ethical leadership (14) Management of the nursing process (16) Giving feedback and rewarding (6) Support to professional development (7) Nursing director (11)	Leadership (7) Working environment (4) Sense of community (4) Requiring factors of the work (8) Participation in decision-making (4) Motivating factors of the work (6) Working welfare (4)	Teamwork within units (4) Teamwork across units (4) Communication openness (3) Handoffs and transitions (4) Supervisor/manager expectations and actions promoting patient safety (4) Management support for patient safety (3) Staffing (4) Organizational learning-continuous improvement (3) Overall perceptions of patient safety (4) Feedback and communication about error (3) Nonpunitive response to error (3) Frequency of events reported (3)	Professional practice (17) Information and participation in own care (11) Cognition of physical needs (4) Human resources (3) Pain and apprehension management (4) Interdisciplinary collaboration (3)
Number of background variables	10	11	8	16

**Table 3:** Transformational leadership, job satisfaction, and patient safety culture scores in 2008-2009 and 2010-2011. Scores range from 1 to 5, with 1 being the lowest and 5 being the highest. Differences between the two survey periods were considered significant if *P* < 0.05.

	Year, *n* mean (SD)	Year, *n* mean (SD)	*P* value
Transformational leadership	2008 (*n* = 499)	2010 (*n* = 498)	
Ethical leadership	3.52 (1.08)	3.63 (1.04)	
Management of the nursing process	3.43 (0.87)	3.43 (0.88)	
Giving feedback and rewarding	3.01 (1.04)	3.07 (1.00)	
Support to professional development	3.72 (0.96)	3.78 (0.97)	
Nursing director	3.01 (0.94)	2.99 (0.91)	
Transformational leadership	3.34 (0.87)	3.39 (0.85)	
Job satisfaction	2008 (*n* = 1176)	2010 (*n* = 779)	
Leadership	3.75 (0.87)	3.86 (0.91)	0.001
Working environment	3.28 (0.88)	3.28 (0.95)	
Sense of community	3.70 (0.74)	3.76 (0.80)	0.030
Requiring factors of the work	3.04 (0.78)	3.16 (0.76)	0.001
Participation in decision-making	3.34 (0.83)	3.44 (0.84)	0.007
Motivating factors of the work	4.23 (0.58)	4.27 (0.60)	0.032
Working welfare	4.14 (0.61)	4.19 (0.57)	0.007
Job satisfaction	3.64 (0.53)	3.71 (0.56)	
Patient safety culture	2008 (*n* = 234)	2011 (*n* = 512)	
Teamwork within units	3.71 (0.69)	3.69 (0.66)	
Teamwork across units	3.53 (0.57)	3.29 (0.63)	<0.001
Communication openness	3.64 (0.61)	3.59 (0.58)	
Handoffs and transitions	3.27 (0.60)	3.03 (0.65)	<0.001
Manager expectations and actions	3.69 (0.73)	3.68 (0.74)	
Management support for patient safety	3.13 (0.79)	3.08 (0.77)	
Staffing	3.19 (0.80)	3.16 (0.85)	
Organizational learning-continuous	3.48 (0.61)	3.42 (0.58)	
improvement			
Overall perceptions of patient safety	3.08 (0.83)	3.14 (0.76)	
Feedback and communication about error	3.14 (0.73)	3.15 (0.70)	
Nonpunitive response to error	3.41 (0.73)	3.48 (0.71)	
Frequency of events reported	3.12 (0.95)	3.26 (0.92)	
Patient safety culture	3.38 (0.46)	3.33 (0.44)	

**Table 4:** Patient satisfaction scores in 2008-2009 and 2010-2011. Scores range from 1 to 5, with 1 being the lowest and 5 being the highest. Differences between the two survey periods were considered significant if *P* < 0.05.

	2008-2009 (*n* = 678), Mean (SD)	2010 (*n* = 867), Mean (SD)	*P* value
Professional practice	4.60 (0.56)	4.62 (0.56)	0.023
Information and participation in own care	4.33 (0.68)	4.37 (0.69)	
Cognition of physical needs	4.41 (0.83)	4.41 (0.87)	
Human resources	3.94 (1.09)	4.06 (1.07)	
Pain and apprehension management	4.05 (0.86)	4.05 (0.89)	
Interdisciplinary collaboration	4.43 (0.74)	4.50 (0.68)	
Patient satisfaction	4.31 (0.62)	4.35 (0.61)	
